# Social isolation and loneliness effects on medication adherence in older adults: perspectives from a systematic review

**DOI:** 10.1186/s12889-025-26085-7

**Published:** 2026-01-28

**Authors:** Alessandra Improta, Sofia Taborri, Noemi Giannetta, Sara Dionisi, Emanuele Di Simone, Erika Renzi, Azzurra Massimi, Aurora De Leo, Nicolò Panattoni, Giovanni Battista Orsi, Fabio Fabbian, Gloria Liquori, Leandro Amato, Marco Di Muzio

**Affiliations:** 1 Department of Biomedicine and Prevention, University of Rome Tor Vergata, Rome, 00133 Italy; 2 Departmental Faculty of Medicine, Saint Camillus International University of Health and Medical Sciences (UniCamillus), Rome, 00131 Italy; 3 Nursing, Technical, Rehabilitation Department, DaTeR Local Health Unit of Bologna, Bologna, 40124 Italy; 4 Department of Medical, Movement and Wellbeing Sciences, Parthenope University of Naples, Naples, 80133 Italy; 5 Department of Public Health and Infectious Diseases, Sapienza University of Rome, Rome, 00185 Italy; 6 Nursing Research Unit IFO, IRCCS Regina Elena National Cancer Institute, Rome, 00144 Italy; 7 Department of Medical Sciences, University of Ferrara, Ferrara, 44121 Italy; 8 Unit of First Aid, Emergency Department, San Filippo Neri Hospital, ASL Roma 1, Rome, Italy; 9 Department of Wellbeing, Health and Environmental Sustainability, Sapienza University of Rome, Rome, 00185 Italy

**Keywords:** Social isolation, Loneliness, Medication adherence, Older adults, Systematic review

## Abstract

**Background:**

Demographic, social, and familial changes have led to an increase in the lack of family and support systems, which is particularly relevant for older adults who may be at risk for low medication adherence. This study examines the relationship between social isolation and/or loneliness and medication adherence in older adults, also identifying the measurement tools used to explore this relationship.

**Methods:**

We conducted a systematic review according to PRISMA guidelines, searching databases including PubMed, CINAHL, PsycINFO, and EBSCO. The quality of evidence was assessed following the GRADE approach, integrated with the JBI Critical Appraisal Tools, NOS for cohort studies and AXIS tools.

**Results:**

In the final synthesis, we included five articles. According to the findings of the study, social isolation and loneliness may negatively affect medication adherence in older adults. Loneliness and social support may have a mediating role in the relationship between social isolation and medication adherence.

**Conclusions:**

Loneliness and social isolation could influence medication adherence in older patients, especially in managing chronic conditions, but more robust research is needed to confirm the associations between social isolation, loneliness, and medication adherence. However, addressing loneliness and social isolation may be critical to improving medication management among older adults.

**Supplementary Information:**

The online version contains supplementary material available at 10.1186/s12889-025-26085-7.

## Background

The older global population, those aged 65 and over [1], is projected to grow from 1 billion in 2020 to 1.4 billion, doubling to 2.1 billion by 2050 [2]. These demographic shifts will result in a significant increase in the demand for healthcare due to age-related challenges [3]. Adults aged 65 and over frequently deal with a wide range of chronic conditions that require multiple and long-term healthcare interventions [4]. Such conditions increase the complexity of managing treatment regimens, including multiple therapies, often leading to polypharmacy, daily habits, lifestyle changes, and involving different healthcare providers [5–7]. For older adults, the correct use of many different drugs over a long period can be highly complex [8, 9]. Medication adherence emerges as a critical public health concern for this population, directly impacting health outcomes [10, 11]. Medication adherence is defined as the process by which patients take their medications and, more generally, follow healthcare providers’ indications as prescribed. This includes the phases of initiation, implementation, and discontinuation [12]. Patients with chronic conditions typically demonstrate lower medication adherence compared to those with acute illnesses, with adherence progressively declining over time [13, 14]. This difference can be attributed to several factors, such as motivational challenges associated with long-term, complex treatment, and the patient’s perception of disease control [15, 16]. Inadequate medication management has substantial consequences, including increased hospital admissions, elevated morbidity, adverse health outcomes, higher mortality rates, and escalated healthcare costs [17–20]. Medication adherence in older adults may be influenced by a complex interplay of personal, health, psychological, and socio-environmental factors [21–23]. Adherence to prescribed medication regimens can be challenging for older adults, especially if they lack family or other support systems [24].

While families have traditionally been the primary source of support for older adults, providing both material and psychological assistance [25, 26], global demographic trends show a decline in family size and an increase in unpartnered middle-aged and older adults with fewer children [27, 28]. Moreover, other relevant transformations that impact support availability include geographic mobility, intergenerational dispersion, and changes in living arrangements [29–31]. Older adults’ lack of adequate or weak, supporting relationships from family, friends, neighbors, or community services [32, 33], age-related factors such as loss of relationships and networks [34], morbidity, functional decline, loss of a partner or friends, financial challenges, and mobility issues [35], make them more vulnerable to social isolation and loneliness than other age groups. Social isolation, understood as the objective reflection of the reduction of the social network, occurs in the absence of relationships or contact with others and little or no social support [36]; this concept is well distinguished from loneliness, which refers to the perception of social isolation or the subjective feeling of being alone, regardless of the number of social contacts [37, 38]. Social isolation and loneliness are two distinct dimensions, representing the objective and subjective aspects of social relationship impoverishment [39].

Based on the previous considerations, it could be hypothesized that objective and subjective aspects of social relationships could fall within the factors influencing medication adherence in older adults. Iovino et al. proposed a middle-range theory, which provides a valuable framework for this hypothesis, elucidating the mechanisms through which impoverished social relationships influence psychological responses, health-related behaviors, and clinical outcomes in individuals with chronic illnesses [40]. According to the theory, social relationship impoverishment is derived from *predisposing* and *precipitating factors* such as age, characteristics of living arrangements, occupational status, stigma, ageism, loss of a significant member, low sense of belonging, physical dysfunction, grief, and sensory deficits [40]; factors to which older adults are particularly exposed.

Given that approximately one in four community-dwelling older adults encounters social isolation, with the global prevalence of this condition within this demographic estimated at 25% [34] and 20.8% experiencing chronic loneliness [41], it becomes crucial to understand the potential influence of social isolation and loneliness on medication adherence among older adults. The frequency and impact of these conditions in this population highlight the importance of understanding the interplay of these factors, which may enhance medication adherence among older adults. Thus, this systematic review investigates the association between social isolation and/or loneliness and medication adherence among older adults aged 65 and above, along with the identification of the measurement tools employed to explore this relationship.

## Methods

According to the revised Preferred Reporting Items for Systematic Reviews and Meta-Analyses (PRISMA) guidelines, a systematic review was conducted [42]. PRISMA checklist is available in the Additional File 1.

The inclusion and exclusion criteria, information sources, and data collection methods were established beforehand.

### Search strategy

The search strategy was developed according to the “PEO” (Population, Exposure, Outcome) framework [43]. This review included quantitative and/or qualitative research focusing on the older population (individuals ≥ 65 years old [1]) or studies where this population has a higher representation in the sample, within the studies evaluated medication adherence and at least one of loneliness or social isolation conditions. Excluded from this review were studies focused on institutionalized/hospitalized patients (considering that healthcare professionals could provide medication management), studies not written in English or Italian, articles unavailable in full text, grey literature, and reviews. No time or geographical restrictions were applied.

The search was conducted among articles published from database inception to March 2025. Related groups of terms (and their variants), were identified as follows: (1) older*, elder*, senior*, aged*, ag?ng; (2) lonel*, “solitud*”, isol*, “social* isolat*”, “social* connect*”, “social contact*”, socialization, “social integrat*”,“social network*”, “social disconnect*”, “social segregat*”, “social interact*” OR “social distan*”; (3) “adherence to treatment*”, “non-adherence”, “non adherence”, “medic* compliance”, “medic* adherence”, “adherence to therapy”. To extend the results, the query string also included terms related to the interest constructs, loneliness, social isolation, and medication adherence. Although the terms “compliance” and “adherence” are conceptually related, each reflects a different perspective: “compliance” implies a more passive patient behavior, while “adherence” emphasizes active and shared involvement in therapeutic decisions [44]. For this reason, “compliance” was not used extensively in the search strategy to avoid including studies that did not align with the construct of adherence adopted in this review.

Identified keywords were combined with Boolean operators, as shown in the table below (Table 1). The resulting query was adapted to PubMed, CINAHL (Cumulative Index to Nursing and Allied Health Literature), PsycINFO, and EBSCO, databases selected – without filters- to find specific biomedical, life sciences, and nursing literature.

**Table 1:** Search strategy Building

PEO approach	Inclusion criteria	Search string
P-Population	Individuals ≥ 65 years old	older* OR elder* OR senior*, aged* OR ag?ng
E-Exposure	Individuals who are socially isolated and/or lonely	lonel* OR “solitud*” OR isol* OR “social* isolat*” OR “social* connect*"OR “social contact*” OR socialization OR “social support*” OR “social network*” OR “social disconnect*” OR “social segregat*” OR “social interact*” OR “social distan*”
O-Outcome	Any qualitative or quantitative measure of medication adherence	“adherence to treatment*” OR “non-adherence” OR “non adherence” OR “medic* compliance” OR “medic* adherence” OR “adherence to therapy”

### Screening, selection, data extraction, and synthesis

Three researchers independently conducted the screening, selection, and data extraction processes, while a fourth researcher resolved any discrepancies disagreements.

Initially, the identified terms were searched for titles and abstracts in each database. After removing duplicates, the initial screening was performed by evaluating the titles and abstracts; subsequently, the selected records were critically examined. For each selected study, the following details were recorded using a pre-constructed form: the first author’s surname and year of publication, study design, sample, objective, studies critical evaluation (risk of bias, inconsistency, indirectness, imprecision, Joanna Briggs Institute (JBI) Checklist Critical Item(s), overall evaluation) (Table 2). The identified measurement instruments to assess social isolation, loneliness, and medication adherence, along with their details, are summarized in an additional table, as shown below (Table 3). Researchers manually extracted data using a shared Excel file table.

**Table 2:** Included studies characteristics and critical evaluation assessment

**Study Characteristics**				**Studies Critical Evaluation**					
**Author** **(years)**	**Study Design**	**Sample**	**Objective**	**Risk of bias**	**Inconsistency**	**Indirectness**	**Imprecision**	**JBI Checklist Critical Item(s)**	**Overall evaluation**
Hacihasanoglu Asilar et al. (2020) [50]	Cross-sectional	397 patients diagnosed with chronic hypertension (mean age 62.64±10.66 SD, with 45.1% of participants being over 65)	Investigating the relationship between loneliness, medication adherence, and self-efficacy in patients with chronic hypertension	serious^abc^	not serious	not serious	not serious	Identification of confounding factors	⨁⨁◯◯ LOW
Lu et al. (2020) [51]	Cross-sectional with integrative theory	2270 older adults with chronic diseases (mean age 70.58±11.05 SD)	Identify the association between social isolation, loneliness, and medication adherence in older adults with chronic disease	serious^b^	not serious	not serious	not serious	----	⨁⨁◯◯ LOW
Sturm et al. (2021) [52]	Cross-sectional study	297 older adults with chronic diseases (mean age 78.46±4.76 SD)	Explore the relationship between psychosocial factors and medication-related beliefs and behaviours	not serious	serious^d^	not serious	not serious	----	⨁⨁⨁◯ MODERATE
Sari et al. (2022)[53]	Cross-sectional study	235 older adults with chronic hypertension (mean age 65.8±8.3 SD)	Examine the impact of feeling lonely and receiving social support on medication adherence in the elderly with hypertension	serious^bc^	not serious	not serious	not serious	Identification of confounding factors	⨁⨁◯◯ LOW
								Description of confounder management strategies	
Yu et al. (2024)[54]	Longitudinal study	797 older adults with chronic disease followed for 1 year (2022–2023) (mean age 69.7±7.9 SD)	Explore the mediating effects and changes of social support and loneliness over time in the association between social isolation and medication adherence	not serious	not serious	not serious	not serious	Similarity between the two groups and recruitment from the same population**	⨁⨁⨁◯ MODERATE

Explanations: a: Sampling method not explicitly declared. b: Response rate omitted. c: Selection bias. d: Social isolation and medication adherence association

SD: Standard Deviation

** Not Applicable

**Table 3:** Loneliness, social isolation and medication adherence measurement tools of the included studies

Variable	Measurement Tools	Description	Studies
Medication Adherence	Morisky Medication Adherence Scale (MMAS-8)	Structured self-report measure composed of 8 items assessing intentional and unintentional medication adherence.	[51, 54]
	Medication Adherence Self-Efficacy Scale Short Form (MASES-SF)	Self-report 13-item instrument, designed to assess perceived medication adherence self-efficacy in hypertensive patients. Each item score ranged from 1 to 4: higher scores indicate better compliance with antihypertensive therapy.	[50]
	Adherence to Refill and Medication Scale (ARMS)	Structured self-report measure consisting of 12 items assessing medication-taking behaviour and prescription refill practices. A higher score indicates lower adherence.	[53]
	Medication Adherence Report Scale (MARS-D)	Structured self-report measure consisting of 5 items assessing medication adherence behaviours and attitudes. The scale evaluates both intentional non-adherence (such as dose adjustments and stopping medication) and unintentional non-adherence (forgetting).	[52]
Social isolation	Social Isolation Index (SII)	5-item instrument. The total score ranges from 0 to 5: a score < 2 indicates a low level of social isolation, while ≥ 2 means a high level of social isolation.	[51, 54]
	Lubben Social Network Scale (LSNS-6)	6-item version. Sum score ranging from 0 to 30: score < 12 indicating risk of social isolation	[52]
Loneliness	UCLA Loneliness Scale (UCLA-LS)	20-item scale. Ten questions are straight-coded, and 10 are reverse-coded. The scale uses a 4-point Likert-type rating: 4 points to “never”, 1 point to “often” for positively worded items and just the opposite for negatively worded items. The total score ranges from 20 to 80. Higher scores indicate higher levels of loneliness.	[50, 53]
	UCLA Loneliness Scale Short-form (ULS-6)	It consists of 6 items scored from 1 “never”, to 4 “often”. Scores ranged from 6 to 24. Higher scores show a higher degree of loneliness.	[51, 54]
	Six-Item (Short) De Jong Gierveld Loneliness Scale (DJG 6-Item)	A six-item instrument measuring through two subscales, emotional and social dimensions of loneliness.	[52]

For each review or any non-primary study detected, authors always made a manual reference screening to capture pertinent research.

Data synthesis was performed through a narrative approach.

### Studies critical evaluation

It was evaluated the quality of the evidence using the Grading of Recommendations Assessment, Development, and Evaluation (GRADE) approach [45]. The evaluation has been integrated using JBI Critical Appraisal Tools: the Checklist for Analytical Cross-sectional Studies and the Checklist for Cohort Studies [46]. The risk of bias has been assessed following the criteria of the Newcastle-Ottawa Scale (NOS) for Cohort Studies [47] and the Appraisal tool for Cross-Sectional Studies (AXIS) [48]. The GRADE domains have been evaluated according to the level of uncertainty (not severe, serious, or severe). Overall certainty has been classified as very low, low, moderate, or high. Certainty was downgraded by one level for each identified limitation in the study design, starting from high certainty. Studies with low certainty exhibit serious risks of bias and data imprecision. Data variability and study quality adversely affected the overall strength of the conclusions.

## Results

**Fig. 1:**
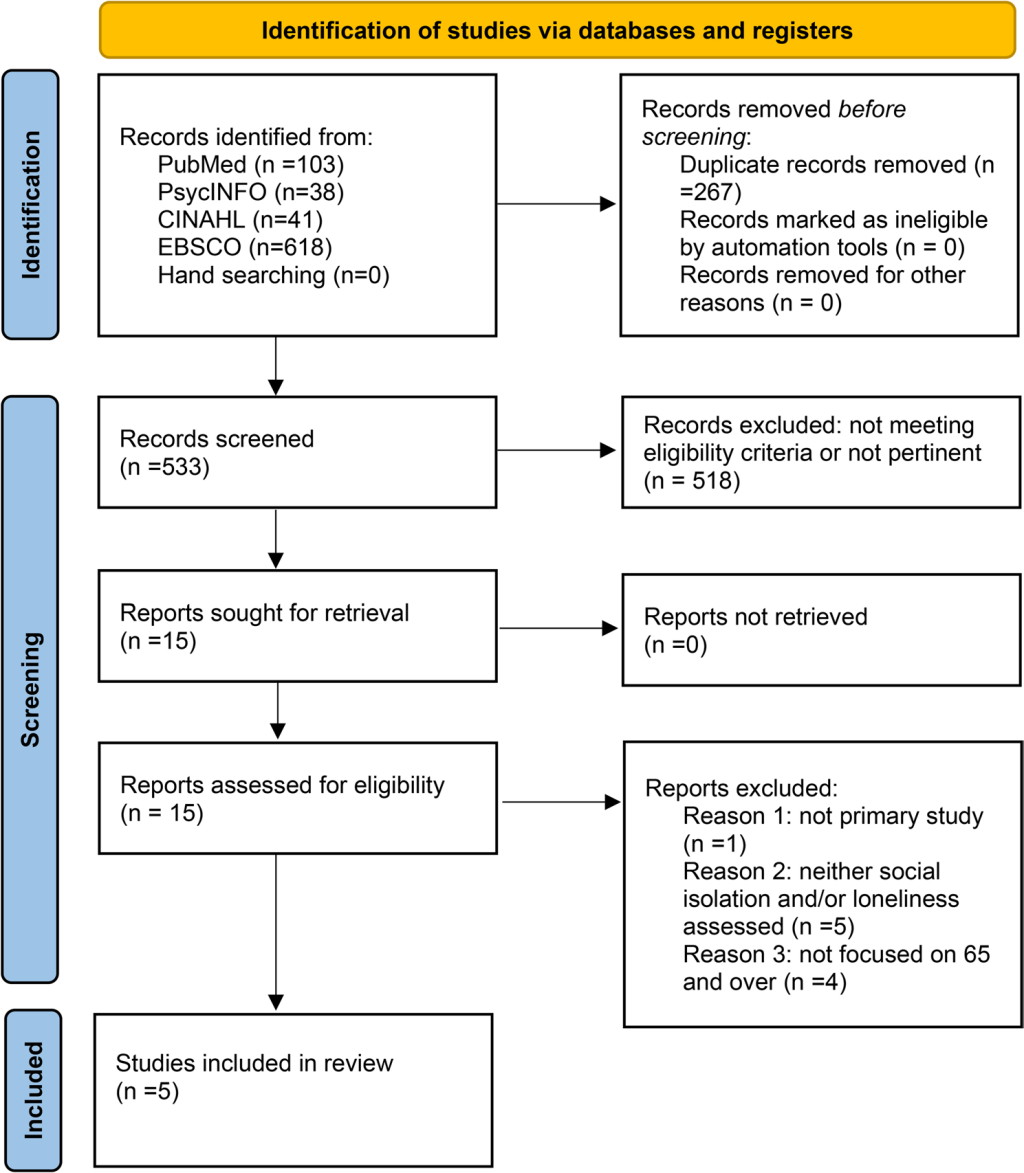
Preferred Reporting Items for Systematic Reviews and Meta-Analyses (PRISMA) 2020 flowchart for study selection

The literature search yielded 800 articles, with 533 remaining after de-duplication. Based on the titles and abstracts, 518 articles have been excluded. The remaining 15 articles were analyzed by full-text reading. Of these, 1 was excluded for design (not being a primary study), 5 for not measuring social isolation or loneliness, and 4 for not focusing on people aged ≥ 65 years. Ultimately, 5 studies met the criteria for eligibility and were included in the review. Figure 1 shows the study’s PRISMA flow chart [49].

### Description of the included studies

The characteristics of the studies included are shown in Table 2. 4 cross-sectional studies [50–53] and 1 longitudinal study [54] were included. Among cross-sectional studies, one has a sample mean age of 62.64 (SD ± 10.66), we included it because adults over 65 are mostly represented (*n* = 179; 45.1%) [50].

Among these studies, 3 were conducted in Asia (China and Indonesia) [51, 53, 54], 1 in Europe (Germany) [52], and 1 in the Middle East (Turkey) [50]. The sample sizes of the studies range from 235 to 2270 participants, with an overall estimated mean age of 69.44 ± 5.97 years. 3 studies [51, 52, 54] explore the interplay between all variables of our interest (loneliness, social isolation, and medication adherence), while 2 studies [50, 53] consider only the relationship between loneliness and medication adherence.

### Effects of social isolation and loneliness on medication adherence

The review findings suggest that loneliness and social isolation are associated with lack of medication adherence in older adults with chronic diseases. Yu et al. revealed a significant decline over time in social support (Social Support Rating Scale: from 26.6 ± 6.2 to 23.5 ± 6.7; *p* < 0.001) and medication adherence (MMAS-8: from 6.7 ± 1.2 to 6.0 ± 1.5; *p* < 0.001) within a significant increase in social isolation (SII: from 1.8 ± 1.3 to 2.5 ± 1.4; *p* < 0.001) and loneliness (ULS-6: 13.2 ± 4.1 to 23.5 ± 6.7; *p* < 0.001): while loneliness and social isolation increased over time, medication adherence declined [54]. Similarly, Lu et al. employing the same instruments, found that cross-sectionally social isolation (*r* = −0.155; *p* < 0.001) and loneliness (*r* = −0.256; *p* < 0.001) were both negatively correlated with medication adherence [51]. Moreover, loneliness and social support seem to mediate the relationship between social isolation and medication adherence in older adults with chronic diseases [51, 54].

Findings do not confirm the relationship between social isolation and medication adherence, as Sturm et al. found a non-statistically significant association between social isolation score (LSNS-6) and medication adherence (MARS-D) (OR = 1.028; *p* = 0.237) [52]. However, this study found that lonely patients (DJG 6-Item) were more likely to be nonadherent to medication (OR = 0.420; *p* < 0.001) [52] while patients who experienced less loneliness were more likely to perceive pharmacological therapy as beneficial to their health (Beliefs about Medicines Questionnaire-General usefulness: rs = − 0.240; *p* < 0.001) [52]. In patients with chronic hypertension, loneliness (UCLA-LS) appears to be correlated with medication adherence (MASES-SF: rs = − 0.287; *p* < 0.001 [50]; ARMS: rs = 0.618; *p* = 0.000 [53]). Hacihasanoglu Asilar and colleagues suggested a significant association between adequate blood pressure control and lower levels of perceived loneliness (UCLA-LS), while no significant correlation was observed between loneliness and factors such as years of hypertension, duration of antihypertensive medication use, or the number of antihypertensive medications used daily [50]. Moreover, Sari et al. revealed that loneliness (UCLA-LS), social support (MOS-Social Support Survey), and medication adherence (ARMS) were correlated (*p* < 0.05), with loneliness emerging as the most significant factor influencing medication adherence in this population [53].

### Measurement tools

The tools used to assess medication adherence included: the Morisky Medication Adherence Scale (MMAS-8) [51, 54], Medication Adherence Report Scale (MARS-D) [52], Adherence to Refill and Medication Scale (ARMS) [53], and Medication Adherence Self-Efficacy Scale Short Form (MASES-SF) [50]. The Social Isolation Index (SII) [51, 54] and Lubben Social Network Scale (LSNS-6) [52], were employed for social isolation assessment. To measure loneliness, researchers utilized 3 different scales: the UCLA Loneliness Scale (UCLA-LS) [50, 53], its Short-form (ULS-6) [51, 54], and the Six-Item (Short) De Jong Gierveld Loneliness Scale (DJG 6-Item) [52]. Table 3 presents the measurement instruments and related characteristics identified through the literature review.

### Critical evaluation of the included studies

All outcomes were classified as moderate [52, 54] or low [50, 51, 53] quality evidence using the GRADE assessment [45], integrated with JBI critical appraisal [46], and following the criteria of the NOS cohort studies [47] and AXIS [48] checklists. It was consistently reduced by one point each for limitations in study design and imprecision. 4 studies have been evaluated through JBI Checklist for Analytical Cross-sectional Studies [50–53] and 1 with the JBI Checklist for Cohort Studies [54]. Risk of bias was assessed in one study using the NOS tool for cohort studies [54] and in 4 studies using the AXIS tool [50–53]. Yu et al. (2024) was rated as not at serious risk of bias after NOS evaluation, with a score of 8/9, with the following scores in each section: *selection* 3, *comparability* 2, *outcome* 3 [54]. In the cross-sectional studies evaluated with the AXIS tool, it was hypothesized a possible selection bias in two studies due to the possible criticism that appeared in the items that examine the sampling process and the representativeness that it ensures (non-random sampling) [50, 53]. Furthermore, one of them doesn’t explicitly report the sampling method [50]. Although Sturm et al. and Yu et al. employed a non-random recruitment method, it was not considered selection bias to be a relevant point of concern, since the recruitment was performed using well-defined inclusion criteria (this ensures representativeness) [52, 54]. The evaluation process then raised concerns about the non-responders rates (and their management) [50, 51, 53]. Concerns have also been raised about the management of confounders in the JBI assessment process [50, 53]. An inconsistency in the results of Sturm et al. [52] has emerged, as this study is the only one to report a non-statistically significant association between social isolation and medication adherence.

The results of the critical appraisal of the included studies are presented in Table 2. Critical evaluation details are available in the Additional File 2.

## Discussion

This systematic review investigated the relationship between social isolation and/or loneliness and medication adherence in older adults while identifying the measurement tools employed to explore this relationship. Despite our findings suggesting the impact of social isolation and loneliness on medication adherence among older adults, limited and heterogeneous evidence pose difficulties in drawing definitive conclusions. In this review, only three studies [51, 52, 54] have concurrently assessed the impact of social isolation and loneliness on medication adherence, and the discrepancies in their findings hinder a comprehensive understanding of the interplay among these variables. Researchers employed various measurement tools, thereby increasing the heterogeneity of results and complicating comparisons. The reviewed studies analyzed loneliness, mostly using the UCLA Loneliness Scale [50, 53] or its abbreviated version [51, 54]. High variability in the choice of instruments was found for measuring medication adherence, with the Morisky Medication Adherence Scale being the most used instrument [51, 54]. While social isolation was mainly assessed using the Social Isolation Index [51, 54]. This measurement disparity made it challenging to compare results, highlighting the need for a more standardized approach to future research in this field. The measures identified in our review are primarily self-report instruments; considering the sensitivity and complexity of the study domains, it is reasonable to expect the results to be susceptible to biases, such as social desirability bias or response bias.

Prior literature identified social isolation as a key factor contributing to nonadherence among adults undergoing chronic therapy [55] and findings from this review appear to corroborate the relationship [51, 54]. However, this result should be interpreted cautiously, as another included study identified a nonsignificant association between social isolation and medication adherence [52]. Two aspects of this study are worthy of note as setting it apart from others: the use of a different instrument for assessing social isolation and the fact that it is the only study conducted in Western society.

Through the lens of middle-range theory [40], social, cultural and environmental contexts are classified as *predisposing factors* that increase the risk of impoverishment of social relationships among people with chronic illnesses [40]. Thus, within the above framework, our findings mainly refer to Eastern societies, which tend to have collectivist cultures that place greater emphasis on family and community, in contrast with Western European cultures, which tend to be more individualistic [56]. Cultural differences can influence the way people perceive relationships. In some patients, having strong ties with the same gender group increases adherence [57]. Future studies should more deeply investigate the *predisposing factors* [40], in particular in the differences in the relationship between social isolation, network composition, and medication adherence among individuals residing in various contexts (e.g., rural vs. urban, villages vs. metropolitan cities) [58], with a deeper analysis of gender differences, and how variations in social networks affect this relationship among older adults. Indeed, the size and the quality of social networks seem to influence adherence [59, 60]. Social networks that provide practical, emotional, and companionship support have been shown to be particularly beneficial [61–63], as patients with greater support are protected from long-term non-adherence [64]. Aligning with the framework considered, the absence of emotional and instrumental support can act as a *precipitating factor* that triggers the deterioration (or the worsening) of social relationships within the chronic illness course: chronically ill patients depend significantly on their social contacts to manage their condition, so insufficient support increases vulnerability to isolation and loneliness and its downstream health consequences [40]. In addition, another important consideration in the study of ageing-related conditions is disability, which, although not explicitly mentioned in the theory, should be considered for the consequent progressive burden of symptoms, impairments, and dysfunctions. Conditions that also act as *precipitating factors* for the objective or subjective deterioration of social relationships, by limiting social interactions [40]. Although the extension of lifespan represents a positive advancement, this does not automatically translate to enhanced well-being: a significant number of older adults with chronic illness face challenges such as loneliness and social isolation [65], with those without care partners facing increased risks of impaired disease self-management, delays in medical treatment, and worse medication adherence [66]. The literature highlights the relationship between social isolation, loneliness, and the incidence and/or progression of disability [67–70]. Considering these factors’ over-time variability is crucial to better understand their significant influence on medication adherence [71].

Statistically significant negative correlations between loneliness and medication adherence emerged from this review [50–52, 54], with only one positive correlation, which can be explained by the medication adherence instrument, where a higher score indicates lower adherence [53]. According to these results, loneliness has also emerged as a mediator in the relationship between social isolation and medication adherence [51, 54]. Still, it has also been found to be a critical factor that affects medication adherence in patients with chronic diseases [50, 53], with older lonely adults who are more likely to be non-adherent compared to their non-lonely counterparts [52]. Following the conceptual framework mentioned, loneliness can directly affect outcomes such as health-related behaviours, or act as a mediator through the proposed mechanism: chronic illnesses increase individuals’ awareness of their need for emotional and instrumental support; the resulting sense of vulnerability may then lead to feelings of loneliness and subsequent consequences for health-related behaviours [40]. High levels of loneliness reduce motivation and willingness to comply with treatments, which are crucial elements for medication adherence [64, 72, 73]; social relationships influence how older adults follow the advice of health professionals [74].

Feelings of loneliness and being socially isolated also have adverse effects on health behaviours, disease self-management (such as not taking medication correctly and disengaging from care), psychological well-being, and health outcomes in older adults dealing with chronic illnesses [75–78]. These elements underscore the importance of incorporating psychosocial factors, particularly loneliness, in interventions to improve medication adherence and overall health outcomes in older patients [52]. Moreover, further investigation may compare different age groups, as social isolation and loneliness affect all population groups, despite older adults’ increased vulnerability [32–35]. Recognizing that social relationships have the potential to influence medication adherence, the scarcity of social ties implies that social isolation and loneliness may constitute factors contributing to nonadherence [24].

Based on the findings of this review, which suggest a possible influence of loneliness and social isolation on medication adherence [50–54], healthcare providers should assess and monitor these factors, along with social support, considering the relationship between isolation and adherence [51, 54]. The results highlight the need to target interventions to prevent and reduce loneliness and social isolation among the older population. Regular screenings to identify these conditions using specific tools, along with the implementation of community-based interventions (such as group activities, internet training, peer support, home visits, or telecare programs), could reduce loneliness and social isolation, strengthen social support, and consequently improve the management of chronic conditions and medication adherence [79, 80]. Nurses play a crucial role in medication management for socially isolated and lonely adults, particularly those with chronic illnesses, given their frequent interaction with older adults and their ability to evaluate and address needs across different levels of care [81]. Systematic implementation of health history interviews, physical examinations, and evidence-based screening tools, as those that emerged from this literature review, should be incorporated routinely [65, 82]. After screening, nurses can further assist older adults who are lonely or socially isolated by connecting them with community resources and integrating psycho-social support into care plans [83]. Expanding social networks and integrating family, community, and organizational involvement are critical elements in enhancing adherence to the treatment regimen [84, 85]. Future research could explore the potential benefits of interventions to prevent or reduce social isolation and loneliness on medication adherence, thereby improving adherence-focused strategies. Interventions should be individualised, considering everyone’s unique circumstances and comorbidities [86]. In addition, adherence behaviour is influenced by contextual factors in which people live, so that interventions may be multilevel, including both individual and contextual factors [87]. Nurses’ involvement in chronic disease management is beneficial when they educate and motivate older adults, support lifestyle changes, provide counselling, and promote behavioural skills and treatment adherence [88, 89]. As the review evidenced, recognising loneliness and social isolation conditions and incorporating these factors into assessing older adults could improve medication adherence and overall health and well-being.

### Limits

This study presents several limitations. First, only five articles met the inclusion criteria, so the evidence is scarce and, moreover, heterogeneous. Firstly, the search strategy was neither validated nor reviewed by a librarian or information specialist, which may have restricted the number of records. Although the search string included terms related to the constructs of interest to improve the search results, the term “compliance” was only used in the context of “medication compliance” rather than in its broader sense (“compliance”). As the term is conceptually related to “adherence”, this approach may have limited the search results. Indeed, we could have placed greater emphasis on the concept of compliance during the search process and then selected results based on the adherence construct followed in this review. Furthermore, the inclusion of studies only in English and Italian languages published in peer-reviewed journals may have resulted in the exclusion of relevant studies that could have been useful for identifying the association between the variables under consideration. Given the inconsistency of findings, particularly regarding the association between social isolation and medication adherence, further studies are necessary to investigate the relationship between these variables. According to the GRADE assessment of the five included studies, the overall certainty of the evidence is low, despite two studies being rated as moderate [52, 54]. Moreover, it has been noted that the results of one study did not align with those of the others [52]. This indicates limited confidence in the synthesis, with a substantial possibility that the true effect differs from the observed findings. Second, the prevalence of cross-sectional studies influences the overall interpretation of the results, as they provide a snapshot of specific populations or contexts, limited to one point in time. This, within the methodological limitations of the sampling strategies used mostly in the included studies, must be considered when extending conclusions to broader populations or different settings. Then, additional studies are needed to assess how these variables change over time and to explore their relationships with one another further. Furthermore, considering that three of five included studies focused on chronic diseases broadly [51, 52, 54], it wasn’t possible to make comparisons about the interplay of variables in older people with specific chronic conditions such as diabetes, chronic obstructive pulmonary disease, or coronary heart disease. Additionally, due to the limited findings, the effects of social isolation and loneliness on medication adherence in older adults could not be explored in terms of the incidence and/or progression of disability.

For this study, older adults were defined as individuals aged 65 years or over, with an average age across the studies of approximately 69 years. Recognizing that the definition of older adults varies between countries and organizations, and that this cut-off is now getting higher in some countries, the inclusion of other cut-offs (e.g., 50 or 60) may have broadened the results from countries with different age-related conditions and life expectancy. Moreover, findings emerged from studies focusing on adults aged 80 and over or age-related analysis may have highlighted results reflecting different experiences, such as social isolation and loneliness, which tend to increase with age. Additionally, the studies utilized different tools to assess medication adherence, loneliness, and social isolation, which exacerbated the heterogeneity of the results. Given the previously mentioned factors, the conclusions presented here should be interpreted with prudence.

## Conclusion

Loneliness and social isolation are prevalent phenomena in the older population that could influence medication adherence, especially in the management of chronic diseases. Although there is limited evidence on the relationship between these phenomena, improving medication adherence in older adults requires an approach that addresses not only the clinical aspects but also the psychological and social ones, such as isolation and loneliness. Due to social and demographic changes, especially in family structure, it is necessary to conduct a global assessment of older adults, focusing on social isolation, loneliness, and potential barriers to social interaction. Considering the results of this review and what is already carried out in the literature, social isolation and loneliness should be considered - and tackled - as real disability, with the same attention as mental or physical ones. Then, considering the topic relevance for public health, promoting strong support networks and addressing loneliness could be effective strategies to optimize adherence to the healthcare providers’ recommendations and prescriptions of older people with chronic diseases. However, more robust research is needed to confirm the associations and mechanisms that link social isolation, loneliness, and medication adherence.

## Supplementary Information

Supplementary Materials: Additional file 1 on Preferred Reporting Items for Systematic Reviews and Meta-Analyses Checklist; Additional file 2 on Critical evaluation of the included studies.

## Data Availability

Data sharing is not applicable to this article as no datasets were generated or analysed during the current study.

## Abbreviations

JBI: Joanna Briggs Institute
NOS: Newcastle Ottawa Scale
GRADE: Grading of Recommendations Assessment, Development, and Evaluation
AXIS: Appraisal tool for Cross-Sectional Studies
PEO: Population, Exposure, Outcome
PRISMA: Preferred Reporting Items for Systematic Reviews and Meta-Analyses
CINAHL: Cumulative Index to Nursing and Allied Health Literature
MMAS-8: Morisky Medication Adherence Scale
MARS-D: Medication Adherence Report Scale
ARMS: Adherence to Refill and Medication Scale
MASES-SF: Medication Adherence Self-Efficacy Scale Short Form
SII: Social Isolation Index
LSNS-6: Lubben Social Network Scale
UCLA-LS: UCLA Loneliness Scale
ULS-6: UCLA Loneliness Scale Short-form
DJG 6-Item: De Jong Gierveld Loneliness Scale Short Form
