# Supplementary material for: Social isolation and loneliness effects on medication adherence in older adults: perspectives from a systematic review
Source: BMC Public Health. 2026 Jan 28;26:577. doi: 10.1186/s12889-025-26085-7 (PMC12896091; doi:10.1186/s12889-025-26085-7)
Supplement: Supplementary file 1 — Supplementary Materials: Additional file 1 on Preferred Reporting Items for Systematic Reviews and Meta-Analyses Checklist; Additional file 2 on Critical evaluation of the included studies. [file 12889_2025_26085_MOESM1_ESM.pdf]

PRISMA

PRISMA 2020 Checklist

Additional File 1

Section and Topic	Item #	Checklist item	Location where item is reported
**TITLE**			
Title	1	Identify the report as a systematic review.	1
**ABSTRACT**			
Abstract	2	See the PRISMA 2020 for Abstracts checklist.	2
**INTRODUCTION**			
Rationale	3	Describe the rationale for the review in the context of existing knowledge.	2-3-4
Objectives	4	Provide an explicit statement of the objective(s) or question(s) the review addresses.	4
**METHODS**			
Eligibility criteria	5	Specify the inclusion and exclusion criteria for the review and how studies were grouped for the syntheses.	4
Information sources	6	Specify all databases, registers, websites, organisations, reference lists and other sources searched or consulted to identify studies. Specify the date when each source was last searched or consulted.	5
Search strategy	7	Present the full search strategies for all databases, registers and websites, including any filters and limits used.	5
Selection process	8	Specify the methods used to decide whether a study met the inclusion criteria of the review, including how many reviewers screened each record and each report retrieved, whether they worked independently, and if applicable, details of automation tools used in the process.	4-5
Data collection process	9	Specify the methods used to collect data from reports, including how many reviewers collected data from each report, whether they worked independently, any processes for obtaining or confirming data from study investigators, and if applicable, details of automation tools used in the process.	6
Data items	10a	List and define all outcomes for which data were sought. Specify whether all results that were compatible with each outcome domain in each study were sought (e.g. for all measures, time points, analyses), and if not, the methods used to decide which results to collect.	5-6
	10b	List and define all other variables for which data were sought (e.g. participant and intervention characteristics, funding sources). Describe any assumptions made about any missing or unclear information.	6
Study risk of bias assessment	11	Specify the methods used to assess risk of bias in the included studies, including details of the tool(s) used, how many reviewers assessed each study and whether they worked independently, and if applicable, details of automation tools used in the process.	6
Effect measures	12	Specify for each outcome the effect measure(s) (e.g. risk ratio, mean difference) used in the synthesis or presentation of results.	Not applicable
Synthesis methods	13a	Describe the processes used to decide which studies were eligible for each synthesis (e.g. tabulating the study intervention characteristics and comparing against the planned groups for each synthesis (item #5)).	6
	13b	Describe any methods required to prepare the data for presentation or synthesis, such as handling of missing summary statistics, or data conversions.	Not applicable
	13c	Describe any methods used to tabulate or visually display results of individual studies and syntheses.	6
	13d	Describe any methods used to synthesize results and provide a rationale for the choice(s). If meta-analysis was performed, describe the model(s), method(s) to identify the presence and extent of statistical heterogeneity, and software package(s) used.	6
	13e	Describe any methods used to explore possible causes of heterogeneity among study results (e.g. subgroup analysis, meta-regression).	Not applicable
	13f	Describe any sensitivity analyses conducted to assess robustness of the synthesized results.	Not applicable
Reporting bias	14	Describe any methods used to assess risk of bias due to missing results in a synthesis (arising from reporting biases).	Not

Section and Topic	Item #	Checklist item	Location where item is reported
assessment			applicable
Certainty assessment	15	Describe any methods used to assess certainty (or confidence) in the body of evidence for an outcome.	6
**RESULTS**			
Study selection	16a	Describe the results of the search and selection process, from the number of records identified in the search to the number of studies included in the review, ideally using a flow diagram.	7
	16b	Cite studies that might appear to meet the inclusion criteria, but which were excluded, and explain why they were excluded.	7
Study characteristics	17	Cite each included study and present its characteristics.	7
Risk of bias in studies	18	Present assessments of risk of bias for each included study.	9-10
Results of individual studies	19	For all outcomes, present, for each study: (a) summary statistics for each group (where appropriate) and (b) an effect estimate and its precision (e.g. confidence/credible interval), ideally using structured tables or plots.	7-8
Results of syntheses	20a	For each synthesis, briefly summarise the characteristics and risk of bias among contributing studies.	7-8-9-10
	20b	Present results of all statistical syntheses conducted. If meta-analysis was done, present for each the summary estimate and its precision (e.g. confidence/credible interval) and measures of statistical heterogeneity. If comparing groups, describe the direction of the effect.	Not applicable
	20c	Present results of all investigations of possible causes of heterogeneity among study results.	Not applicable
	20d	Present results of all sensitivity analyses conducted to assess the robustness of the synthesized results.	Not applicable
Reporting biases	21	Present assessments of risk of bias due to missing results (arising from reporting biases) for each synthesis assessed.	Not applicable
Certainty of evidence	22	Present assessments of certainty (or confidence) in the body of evidence for each outcome assessed.	10
**DISCUSSION**			
Discussion	23a	Provide a general interpretation of the results in the context of other evidence.	10-11-12-13
	23b	Discuss any limitations of the evidence included in the review.	14-15
	23c	Discuss any limitations of the review processes used.	14-15
	23d	Discuss implications of the results for practice, policy, and future research.	13
**OTHER INFORMATION**			
Registration and protocol	24a	Provide registration information for the review, including register name and registration number, or state that the review was not registered.	Not applicable
	24b	Indicate where the review protocol can be accessed, or state that a protocol was not prepared.	Not applicable
	24c	Describe and explain any amendments to information provided at registration or in the protocol.	Not applicable
Support	25	Describe sources of financial or non-financial support for the review, and the role of the funders or sponsors in the review.	16

Section and Topic	Item #	Checklist item	Location where item is reported
Competing interests	26	Declare any competing interests of review authors.	16
Availability of data, code and other materials	27	Report which of the following are publicly available and where they can be found: template data collection forms; data extracted from included studies; data used for all analyses; analytic code; any other materials used in the review.	15

From: Page MJ, McKenzie JE, Bossuyt PM, Boutron I, Hoffmann TC, Mulrow CD, et al. The PRISMA 2020 statement: an updated guideline for reporting systematic reviews. BMJ 2021;372:n71. doi: 10.1136/bmj.n71.
This work is licensed under CC BY 4.0. To view a copy of this license, visit https://creativecommons.org/licenses/by/4.0/

Additional File 2

Appraisal tool for Cross-Sectional Studies (AXIS)

Paper: Hacihasanoglu Asilar et al. (2020)

ID Number: 50

Appraisal of Cross-sectional Studies

	Question	Yes	No	Don't know/ Comment
	Introduction			
1	Were the aims/objectives of the study clear?	X		
	Methods			
2	Was the study design appropriate for the stated aim(s)?	X		
3	Was the sample size justified?	X		
4	Was the target/reference population clearly defined? (Is it clear who the research was about?)	X		
5	Was the sample frame taken from an appropriate population base so that it closely represented the target/reference population under investigation?	X		
6	Was the selection process likely to select subjects/participants that were representative of the target/reference population under investigation?			X authors declare :" No sampling was attempted for the study"
7	Were measures undertaken to address and categorise non-responders?		X	
8	Were the risk factor and outcome variables measured appropriate to the aims of the study?	X		
9	Were the risk factor and outcome variables measured correctly using instruments/measurements that had been trialled, piloted or published previously?	X		UCLA-LS, the Medication Adherence Self-Efficacy Scaled Short Form
10	Is it clear what was used to determined statistical significance and/or precision estimates? (e.g. p-values, confidence intervals)	X		
11	Were the methods (including statistical methods) sufficiently described to enable them to be repeated?	X		
	Results			
12	Were the basic data adequately described?	X		
13	Does the response rate raise concerns about non-response bias?			X response rate omitted
14	If appropriate, was information about non-responders described?			X
15	Were the results internally consistent?	X		
16	Were the results presented for all the analyses described in the methods?	X		
	Discussion			
17	Were the authors' discussions and conclusions justified by the results?	X		
18	Were the limitations of the study discussed?	X		
	Other			
19	Were there any funding sources or conflicts of interest that may affect the authors' interpretation of the results?		X	
20	Was ethical approval or consent of participants attained?	X		

Additional File 2

Paper: Lu et al. (2020)

ID Number: 51

Appraisal of Cross-sectional Studies

	Question	Yes	No	Don't know/ Comment
	Introduction			
1	Were the aims/objectives of the study clear?	X		
	Methods			
2	Was the study design appropriate for the stated aim(s)?	X		
3	Was the sample size justified?	X		
4	Was the target/reference population clearly defined? (Is it clear who the research was about?)	X		
5	Was the sample frame taken from an appropriate population base so that it closely represented the target/reference population under investigation?	X		
6	Was the selection process likely to select subjects/participants that were representative of the target/reference population under investigation?	X		stratified random cluster sampling
7	Were measures undertaken to address and categorise non-responders?		X	
8	Were the risk factor and outcome variables measured appropriate to the aims of the study?	X		MCIRS-G, MMAS-8, five-item index of social isolation, ULS-6, MOS-SSS
9	Were the risk factor and outcome variables measured correctly using instruments/measurements that had been trialled, piloted or published previously?	X		
10	Is it clear what was used to determined statistical significance and/or precision estimates? (e.g. p-values, confidence intervals)	X		
11	Were the methods (including statistical methods) sufficiently described to enable them to be repeated?	X		
	Results			
12	Were the basic data adequately described?	X		
13	Does the response rate raise concerns about non-response bias?			X<br>Response rate omitted
14	If appropriate, was information about non-responders described?			X
15	Were the results internally consistent?	X		
16	Were the results presented for all the analyses described in the methods?	X		
	Discussion			
17	Were the authors' discussions and conclusions justified by the results?	X		

18	Were the limitations of the study discussed?	X		
Other				
19	Were there any funding sources or conflicts of interest that may affect the authors' interpretation of the results?		X	
20	Was ethical approval or consent of participants attained?	X		

Paper: Sturm et al. (2021)

ID Number: 52

Appraisal of Cross-sectional Studies

	Question	Yes	No	Don't know/ Comment
Introduction				
1	Were the aims/objectives of the study clear?	X		
Methods				
2	Was the study design appropriate for the stated aim(s)?	X		
3	Was the sample size justified?	X		
4	Was the target/reference population clearly defined? (Is it clear who the research was about?)	X		
5	Was the sample frame taken from an appropriate population base so that it closely represented the target/reference population under investigation?	X		
6	Was the selection process likely to select subjects/participants that were representative of the target/reference population under investigation?	X		Not a random sample, but recruitment was done by physicians using well defined criteria to select patients for a trial.
7	Were measures undertaken to address and categorise non-responders?		X	
8	Were the risk factor and outcome variables measured appropriate to the aims of the study?	X		
9	Were the risk factor and outcome variables measured correctly using instruments/measurements that had been trialled, piloted or published previously?	X		MARS-D, BMQ, GSE, SES6G, SpNQ-20, PAM13-D, DJG 6-item, LSNS-6
10	Is it clear what was used to determined statistical significance and/or precision estimates? (e.g. p-values, confidence intervals)	X		
11	Were the methods (including statistical methods) sufficiently described to enable them to be repeated?	X		

Results				
12	Were the basic data adequately described?	X		
13	Does the response rate raise concerns about non-response bias?		X	*"323 patients had given consent to participate in the study. Of those, 297 completed the baseline assessment and were therefore included in the intention-to-treat analysis of the HoPES3 study. The same population was chosen for this explorative cross-sectional study."*
14	If appropriate, was information about non-responders described?	-	-	
15	Were the results internally consistent?	X		
16	Were the results presented for all the analyses described in the methods?	X		
Discussion				
17	Were the authors' discussions and conclusions justified by the results?	X		
18	Were the limitations of the study discussed?	X		
Other				
19	Were there any funding sources or conflicts of interest that may affect the authors' interpretation of the results?		X	
20	Was ethical approval or consent of participants attained?	X		

Paper: Sari et al. (2022)
ID Number: 53

Appraisal of Cross-sectional Studies

	**Question**	**Yes**	**No**	**Don't know/ Comment**
Introduction				
1	Were the aims/objectives of the study clear?	X		
Methods				
2	Was the study design appropriate for the stated aim(s)?	X		
3	Was the sample size justified?	X		
4	Was the target/reference population clearly defined? (Is it clear who the research was about?)	X		
5	Was the sample frame taken from an appropriate population base so that it closely represented the target/reference population under investigation?	x		

6	Was the selection process likely to select subjects/participants that were representative of the target/reference population under investigation?			X (non-random sampling method)
7	Were measures undertaken to address and categorise non-responders?		X	
8	Were the risk factor and outcome variables measured appropriate to the aims of the study?	X		
9	Were the risk factor and outcome variables measured correctly using instruments/measurements that had been trialled, piloted or published previously?	X		UCLA-Loneliness Scale, MOS-Social Support Survey, and ARMS scale
10	Is it clear what was used to determined statistical significance and/or precision estimates? (e.g. p-values, confidence intervals)	X		
11	Were the methods (including statistical methods) sufficiently described to enable them to be repeated?	X		
	Results			
12	Were the basic data adequately described?	X		
13	Does the response rate raise concerns about non-response bias?			X Response rate omitted
14	If appropriate, was information about non-responders described?		X	
15	Were the results internally consistent?	X		
16	Were the results presented for all the analyses described in the methods?	X		
	Discussion			
17	Were the authors' discussions and conclusions justified by the results?	X		
18	Were the limitations of the study discussed?	X		
	Other			
19	Were there any funding sources or conflicts of interest that may affect the authors' interpretation of the results?		X	
20	Was ethical approval or consent of participants attained?	X		

NEWCASTLE - OTTAWA QUALITY ASSESSMENT SCALE

PAPER: Yong Yu, 2024 **ID Number**: 54

COHORT STUDIES

<u>Note</u>: A study can be awarded a maximum of one star for each numbered item within the Selection and Outcome categories. A maximum of two stars can be given for Comparability

Selection

1) <u>Representativeness of the exposed cohort</u>
   a) truly representative of the average _*older adults with chronic conditions*___in the community
   b) somewhat representative of the average _ *older adults with chronic conditions* (*non-random sampling*)______in the community ❋
   c) selected group of users eg nurses, volunteers
   d) no description of the derivation of the cohort

2) <u>Selection of the non exposed cohort</u>
   a) drawn from the same community as the exposed cohort
   b) drawn from a different source
   c) no description of the derivation of the non exposed cohort

3) <u>Ascertainment of exposure</u>
   a) secure record (eg surgical records)
   b) structured interview
   c) written self report ❋
   d) no description

4) <u>Demonstration that outcome of interest was not present at start of study</u>
   a) yes ❋
   b) no

Comparability

1) <u>Comparability of cohorts on the basis of the design or analysis</u>
   a) study controls for _*sociodemographic and clinical covariates* (in the statistical model)________ (select the most important factor) ❋
   b) study controls for any additional factor _*social support and loneliness* (mediators of the relationship between social isolation and medication adherence)______❋ (This criteria could be modified to indicate specific control for a second important factor.)

Outcome

1) <u>Assessment of outcome</u>
   a) independent blind assessment
   b) record linkage
   c) self report ❋
   d) no description

2) <u>Was follow-up long enough for outcomes to occur</u>
   a) yes (follow up length: 1 year) ❋
   b) no

3) Adequacy of follow up of cohorts

a) complete follow up - all subjects accounted for

b) subjects lost to follow up unlikely to introduce bias - small number lost - < 20% _(*2.8%)*____ (select an adequate %) follow up, or description provided of those lost) ❋

c) follow up rate < ____% (select an adequate %) and no description of those lost

d) no statement

Rating:

**Selection**	**Comparability**	**Outcome**
❋❋❋	❋❋	❋❋❋

Coding

- **Exposed Cohort**: 797 older adults with chronic conditions recruited from a regional hospital
- **Exposure**: social isolation
- **Outcome**: medication adherence (MMAS-8 instrument)
- **Follow up**:
  - rate 97.2%
  - lost: 2.8% (health problems)

Additional File 2

JBI CRITICAL APPRAISAL CHECKLIST FOR ANALYTICAL CROSS SECTIONAL STUDIES

Reviewer_________AI_________________________________Date____May 2025, 25_____________

Author_______Hacihasanoglu Asilar et al.___________Year_2020__ Record Number_39______

	Yes	No	Unclear	Not applicable
1. Were the criteria for inclusion in the sample clearly defined?	☒	☐	☐	☐
2. Were the study subjects and the setting described in detail?	☒	☐	☐	☐
3. Was the exposure measured in a valid and reliable way?	☒	☐	☐	☐
4. Were objective, standard criteria used for measurement of the condition?	☒	☐	☐	☐
5. Were confounding factors identified?	☐	☐	☒	☐
6. Were strategies to deal with confounding factors stated?	☒	☐	☐	☐
7. Were the outcomes measured in a valid and reliable way?	☒	☐	☐	☐
8. Was appropriate statistical analysis used?	☒	☐	☐	☐

Overall appraisal: Include ☒ Exclude ☐ Seek further info ☐

Comments (Including reason for exclusion)

____

____

____

____

JBI CRITICAL APPRAISAL CHECKLIST FOR ANALYTICAL CROSS SECTIONAL STUDIES

Reviewer____________AI_________________________________ Date_______May 2025, 25____________

Author_____Jiao Lu____________________________Year_2020________ Record Number_40____

	Yes	No	Unclear	Not applicable
1. Were the criteria for inclusion in the sample clearly defined?	☒	☐	☐	☐
2. Were the study subjects and the setting described in detail?	☒	☐	☐	☐
3. Was the exposure measured in a valid and reliable way?	☒	☐	☐	☐
4. Were objective, standard criteria used for measurement of the condition?	☒	☐	☐	☐
5. Were confounding factors identified?	☒	☐	☐	☐
6. Were strategies to deal with confounding factors stated?	☒	☐	☐	☐
7. Were the outcomes measured in a valid and reliable way?	☒	☐	☐	☐
8. Was appropriate statistical analysis used?	☒	☐	☐	☐

Overall appraisal: Include ☒ Exclude ☐ Seek further info ☐

Comments (Including reason for exclusion)

____

____

____

JBI CRITICAL APPRAISAL CHECKLIST FOR ANALYTICAL CROSS SECTIONAL STUDIES

Reviewer_______________AI____________________________Date______May 2025, 25______________

Author______Sturm et al.______________________Year_2021_________ Record Number__41_______

	Yes	No	Unclear	Not applicable
1. Were the criteria for inclusion in the sample clearly defined?	☒	☐	☐	☐
2. Were the study subjects and the setting described in detail?	☒	☐	☐	☐
3. Was the exposure measured in a valid and reliable way?	☒	☐	☐	☐
4. Were objective, standard criteria used for measurement of the condition?	☒	☐	☐	☐
5. Were confounding factors identified?	☒	☐	☐	☐
6. Were strategies to deal with confounding factors stated?	☒	☐	☐	☐
7. Were the outcomes measured in a valid and reliable way?	☒	☐	☐	☐
8. Was appropriate statistical analysis used?	☒	☐	☐	☐

Overall appraisal: Include ☒ Exclude ☐ Seek further info ☐

Comments (Including reason for exclusion)

____

____

____

JBI CRITICAL APPRAISAL CHECKLIST FOR ANALYTICAL CROSS SECTIONAL STUDIES

Reviewer___________AI_________________________________Date____May 2025, 25___________

Author _Dwi Novita Sari__________Year_2022______ Record Number_42______

	Yes	No	Unclear	Not applicable
1. Were the criteria for inclusion in the sample clearly defined?	☒	☐	☐	☐
2. Were the study subjects and the setting described in detail?	☒	☐	☐	☐
3. Was the exposure measured in a valid and reliable way?	☒	☐	☐	☐
4. Were objective, standard criteria used for measurement of the condition?	☒	☐	☐	☐
5. Were confounding factors identified?	☐	☐	☒	☐
6. Were strategies to deal with confounding factors stated?	☐	☐	☒	☐
7. Were the outcomes measured in a valid and reliable way?	☒	☐	☐	☐
8. Was appropriate statistical analysis used?	☒	☐	☐	☐

Overall appraisal: Include ☒ Exclude ☐ Seek further info ☐

Comments (Including reason for exclusion)

____

____

____

JBI CRITICAL APPRAISAL CHECKLIST FOR COHORT STUDIES

Reviewer________________AI____________________________Date______May 2025, 25__________

Author Yong Yu_____________________ Year_2024________ Record Number___43_______

	Yes	No	Unclear	Not applicable
1. Were the two groups similar and recruited from the same population?	☐	☐	☐	☒
2. Were the exposures measured similarly to assign people to both exposed and unexposed groups?	☐	☐	☐	☒
3. Was the exposure measured in a valid and reliable way?	☒	☐	☐	☐
4. Were confounding factors identified?	☒	☐	☐	☐
5. Were strategies to deal with confounding factors stated?	☒	☐	☐	☐
6. Were the groups/participants free of the outcome at the start of the study (or at the moment of exposure)?	☒	☐	☐	☐
7. Were the outcomes measured in a valid and reliable way?	☒	☐	☐	☐
8. Was the follow up time reported and sufficient to be long enough for outcomes to occur?	☒	☐	☐	☐
9. Was follow up complete, and if not, were the reasons to loss to follow up described and explored?	☒	☐	☐	☐
10. Were strategies to address incomplete follow up utilized?	☒	☐	☐	☐
11. Was appropriate statistical analysis used?	☒	☐	☐	☐

Overall appraisal: Include ☒ Exclude ☐ Seek further info ☐

Comments (Including reason for exclusion)

____

____
